# Ferritin Light Chain Confers Protection Against Sepsis-Induced Inflammation and Organ Injury

**DOI:** 10.3389/fimmu.2019.00131

**Published:** 2019-02-04

**Authors:** Abolfazl Zarjou, Laurence M. Black, Kayla R. McCullough, Travis D. Hull, Stephanie K. Esman, Ravindra Boddu, Sooryanarayana Varambally, Darshan S. Chandrashekar, Wenguang Feng, Paolo Arosio, Maura Poli, Jozsef Balla, Subhashini Bolisetty

**Affiliations:** ^1^Department of Medicine, University of Alabama at Birmingham, Birmingham, AL, United States; ^2^Nephrology Research and Training Center, University of Alabama at Birmingham, Birmingham, AL, United States; ^3^Department of Pathology, University of Alabama at Birmingham, Birmingham, AL, United States; ^4^Department of Molecular and Translational Medicine, University of Brescia, Brescia, Italy; ^5^Department of Nephrology, Faculty of Medicine, University of Debrecen, Debrecen, Hungary; ^6^Department of Cell, Development and Integrative Biology, University of Alabama at Birmingham, Birmingham, AL, United States

**Keywords:** ferritin, inflammatory response, sepsis, NF-κB, cytokine, LPS, multi-organ injury, myeloid cells

## Abstract

Despite the prevalence and recognition of its detrimental impact, clinical complications of sepsis remain a major challenge. Here, we investigated the effects of myeloid ferritin heavy chain (FtH) in regulating the pathogenic sequelae of sepsis. We demonstrate that deletion of myeloid FtH leads to protection against lipopolysaccharide-induced endotoxemia and cecal ligation and puncture (CLP)-induced model of sepsis as evidenced by reduced cytokine levels, multi-organ dysfunction and mortality. We identified that such protection is predominantly mediated by the compensatory increase in circulating ferritin (ferritin light chain; FtL) in the absence of myeloid FtH. Our *in vitro* and *in vivo* studies indicate that prior exposure to ferritin light chain restrains an otherwise dysregulated response to infection. These findings are mediated by an inhibitory action of FtL on NF-κB activation, a key signaling pathway that is implicated in the pathogenesis of sepsis. We further identified that LPS mediated activation of MAPK pathways, specifically, JNK, and ERK were also reduced with FtL pre-treatment. Taken together, our findings elucidate a crucial immunomodulatory function for circulating ferritin that challenges the traditional view of this protein as a mere marker of body iron stores. Accordingly, these findings will stimulate investigations to the adaptive nature of this protein in diverse clinical settings.

## Introduction

Sepsis is a severe, dynamic, and deranged immune response to an infectious insult (1, 2). The complexity of this clinical syndrome is perhaps best highlighted by its evolving definitions (3, 4). The most recent international consensus defines sepsis as life-threatening organ dysfunction resulting from a dysregulated host response to infection (4). The overall mortality rates of sepsis are alarmingly high and range from 10 to 50% of diagnosed patients with development of organ injury and septic shock as two major factors leading to higher mortality rates (5–7). Consequently, the massive burden of morbidity, mortality, and healthcare expenditure associated with sepsis underscore the desperate need for gaining more insight into this devastating clinical syndrome and identifying novel targets for prevention and therapy. Discovery of penicillin by Sir Alexander Fleming was a major defining moment in human history and its struggle against infections. Nevertheless, despite vigorous investigations, overall improvement in our understanding of the molecular mechanisms involved in the pathogenesis of sepsis and numerous clinical trials, an “anti-sepsis” drug remains elusive (8, 9).

Ferritin is a spherical protein made of 24 subunits (10). These subunits are composed of heavy (FtH) and light (FtL) chains and their proportional contribution to the hollow spherical shell varies among tissues. Ferritin was traditionally considered to be a cytosolic protein with the mere function of iron storage. However, our understanding of this highly evolutionarily-preserved molecule has dramatically advanced in the past couple of decades. It is now clear that within the subcellular compartments, ferritin is present in the mitochondria and nucleus (11–13). Additionally, several functions other than iron storage including immunomodulatory attributes have been described [reviewed in (14, 15)]. Another interesting aspect of ferritin in the context of biological activity relates to serum ferritin. The precise source of serum ferritin was long debated. However, in an elegant study Cohen et al. revealed that serum ferritin is mainly a secreted protein, involving a non-classical lysosomal pathway (16). Furthermore, it was shown that FtL is the main form of circulatory protein, its iron content is low and more importantly, macrophages are the main source of serum ferritin (16). Serum ferritin level has been utilized by clinicians as part of a panel to assess iron stores. However, serum ferritin is also increased in response to infections, inflammation and malignancy and is generally accepted to be an acute phase reactant similar to C-reactive protein (17, 18). Despite this knowledge and our evolving understanding of the well-orchestrated pathways involved in cellular and systemic iron homeostasis, the precise role of serum ferritin and its impact on disease progression is yet to be conspicuously defined. While the connotation of iron's role in infection is extensively debated, lack of consistent evidence precludes us to comprehensively define the paradigm of such relationship and more importantly how serum ferritin may be involved in this context. Here, we asked whether myeloid cell specific FtH deletion modulates inflammation and organ injury in a commonly used and well-established model of poly-microbial sepsis, cecal ligation and puncture (CLP).

## Materials and Methods

### Experimental Model and Subject Details

Male and female H-ferritin floxed mice (FtH^fl/fl^) and myeloid-specific H-ferritin deletion mice (FtH^LysM−/−^) (10–14 weeks of age), previously described (19, 20), were used in this study. FtH^fl/fl^ mice were generated on a mixed background (C57BL/6 × 129/Sv) and were backcrossed to C57BL/6 for 10 generations. LyzM^Cre^ mice were generated on a mixed background (Sv129 × C57BL/6 × CB.20) but were backcrossed to a C57BL/6 background for six generations before submission to Jackson Laboratories (Jackson Laboratories; stock 004781). Myeloid-specific FtH deletion mice on a C57BL/6 background were obtained by breeding FtH^fl/fl^ and LyzM^Cre^ mice. All mice were treated humanely and methods were approved by the Institutional Animal Care and Use Committee of UAB. Studies were performed in accordance with National Institutes of Health guidelines.

### Cecal Ligation and Puncture-Induced Sepsis

Mice were anesthetized with isoflurane (1.5–2% isoflurane induction, 1–1.5% maintenance). Midline laparotomy incision (1–2 cm) was made under aseptic conditions to expose the cecum, and stool was milked to the tip and ligated 0.7 centimeter from the tip. Using a 21G needle, two punctures were made in the cecal wall and fecal material was expressed in to the peritoneal cavity. The cecum was placed back in the peritoneal cavity and musculature and skin were sutured. Sham animals underwent laparotomy and bowel manipulation without ligation or perforation. Mice were placed on a heating pad for recovery and provided unrestricted access to water and food. Mice were monitored closely for signs and symptoms of pain and distress (usually developed 6–12 h after surgery). Mice were sacrificed 8 and 24 h after surgery. Serum was collected for creatinine measurement by LC-MS/MS. Endotoxin-free recombinant FtL (1 mg per 25 g B.W.) was administered intravenously 24 h prior to CLP surgery.

### Lipopolysaccharide (LPS)-Induced Sepsis

Mice were anesthetized with isoflurane 1.5–2% isoflurane induction, 1–1.5% maintenance and injected intraperitoneally with 8 mg/kg LPS (Invivogen) and were sacrificed 24 h after injection. Serum was collected for creatinine measurement by LC-MS/MS.

### Radiotelemetric Blood Pressure Measurement

Blood pressure was recorded and analyzed via left carotid artery PA-C10 telemetry implant (DSI, Saint Paul, MN), as previously described (21, 22). Sepsis was induced by CLP after baseline measurements were performed and blood pressure was monitored. The device implantation and monitoring were performed by the UAB-UCSD O'Brien Center Core Facility.

### Transcutaneous GFR Measurement

Transcutaneous GFR was measured in mice using fluorescein isothocyanate (FITC)-labeled sinistrin (MediBeacon), as previously described (23). GFR was monitored for 2 h, after which the monitors were removed and data were analyzed using elimination kinetics curve of sinistrin clearance, as previously described (24).

### Measurement of Serum Cytokine Levels

Mouse serum cytokine levels were measured using mouse V-PLEX Pro-inflammatory Panel I Kit (Meso Scale Discovery), following manufacturers recommendations. Data was acquired using a MESO Sector S600 plate reader (Meso Scale Discovery). Cytokine levels were represented as picograms or nanograms per milliliter.

### Flow Cytometry

Mice were anesthetized with 1.5–2% isoflurane and blood was collected using cardiac puncture. Organs were perfused with cold PBS and spleen was harvested and weighed. Flow cytometry was performed as described previously (20, 25). Following antibodies (with their respective clone numbers) were obtained from eBioscience unless otherwise stated: 7-AAD, MHCII-FITC (M5/114.15.2), Gr-1-APC (1A8), Ly6C-eF450 (HK1.4), CD11b-SuperBright600 (M1/70), CD45.2-BV650 (104; BioLegend), F4/80-APC-eF780 (BM8), CD11c-BV785 (N418; BioLegend), NK1.1-PE (PK136), CD3e-APC (145-2C11), CD8a-eF450 (53-6.7), CD4-SuperBright600 (RM4-5), CD19-BV785 (6D5; BioLegend). AccuCheck beads (Life Technologies) were used to determine absolute numbers by normalizing to tissue mass. Data were collected with a Becton-Dickinson LSRII analyzer and analyzed using FlowJo software (Trecstar Software).

### Peritoneal Colony Forming Unit (CFU) Measurement

Mice were anesthetized with 1.5–2% isoflurane and peritoneal lavage was sterilely collected in 0.9% sterile saline. Serial dilutions of the peritoneal lavage fluid were made and plated on Trypticase Soy Agar with Sheep Blood (Fisher Scientific). Agar plates were incubated at 37°C for 24 h. Single colonies were counted and data were represented as CFU per milliliter.

### *In vitro* Phagocytosis

Bone marrow-derived macrophages (BMDMs) were changed to DMEM + 1% FBS media 24 h before being treated with LPS (100 ng/mL) or Apoferritin (0.1 mg/mL; Sigma). BMDMs were harvested and stained with CD11b-APC (M1/70; eBioscience), 7AAD (eBioscience). Cells were simultaneously incubated with MOI 10 pHrodo Red *Escherichia coli* BioParticles (ThermoFisher), as previously described with minor modifications (26). Cells were analyzed on a BD FACSCalibur and data was analyzed using FlowJo software.

### Bacterial Propagation

Xen14 (bioluminescent *Escherichia coli* WS2572 parent strain, Perkin Elmer) was cultured overnight in an orbital shaker at 200 rotations per minute at 37°C in Luria Broth medium containing 30 μg/mL kanamycin (Fisher Scientific). Initial absorbance was determined at 600 nm and dilutions were prepared with antibiotic-free DMDM + 1% FBS for all experiments.

### *In vitro* Bacterial Killing

Bone marrow-derived macrophages (BMDMs) were changed to DMEM + 1% FBS media 24 h before this assay. Absorbance of cultured Xen14 was determined at 600 nm and dilutions were prepared in antibiotic-antimycotic free cell culture media. BMDMs were treated with MOI 10 or 100 for 30 min. Bacterial media was aspirated and BMDMs were washed with PBS. DMEM + 1% FBS + 200 μg/mL gentamicin was added for 2 h and luminescence quantified on a Biotek Synergy HT plate reader. Data were expressed as arbitrary units (AU) of luminescence.

### *In vivo* Bacterial Killing

Absorbance of cultured Xen14 was determined and dilutions were prepared in 0.9% sterile normal saline. The abdomen of the mice was shaved and injected intraperitoneally with 10^6^ Xen14. Mice were immediately imaged using a Xenogen IVIS-50 Bioluminescence Reader, housed by the UAB Preclinical Imaging Shared Facility. Mice were imaged at 2, 4, 8, and 24 h after injection. Data were expressed as mean fluorescence index in AU.

### Cecal Slurry Preparation

Cecal slurry was collected from male wild-type mice, as previously described (27). Equal volume of 30% glycerol in sterile PBS was added, then cecal slurry was aliquoted in to cryovials for storage at −80°C.

### Purification of Recombinant Human FtL

Recombinant human FtL was generated as previously described (28). Briefly, FtL was expressed in *E. coli* using a pDS20pTrp vector. Gel purification was performed on a Sepharose 6B column, followed by a DEAE column. Endotoxin was removed using Pierce High Capacity Endotoxin Removal Spin Columns (ThermoFisher), per manufacturer protocol. Electrophoretic purity was determined (96% pure) and removal of endotoxin was confirmed using an E-Toxate kit (Sigma). Apoferritin derived from equine spleen was bought from Sigma (A3641).

### Cell Culture

Bone marrow-derived macrophages (BMDMs) were isolated from FtH^fl/fl^ and FtH^LysM−/−^ mice, as previously described, with minor modifications (20). BMDMs were cultured for 6 days before experiments were performed. BMDMs were plated onto culture dishes and treated with L-ferritin, or Apoferritin (0.1 mg/ml) for 16 h. Media was removed, the cells were washed with PBS and treated with LPS (100 ng/mL) or cecal slurry (1 μL per one million cells) in DMEM + 1% FBS for the indicated times. Cells were then washed with PBS and collected for RNA or protein analysis.

### Quantification of mRNA Expression

Gene expression analysis was performed, as previously described (29). Primers used to detect the specific genes are listed in Table S4. For the RNA sequencing studies, total RNA was isolated from blood via cardiac puncture using a Blood RNA isolation kit (ThermoFisher Scientific) and subsequently, globin mRNA was depleted using GLOBINclear kit (ThermoFisher Scientific). RNA was sequenced on NextSeq500 system and the library was prepared with the Agilent SureSelect Stranded mRNA kit.

### Western Blot

All western blotting was performed as previously described (20). Membranes were blocked according to manufacturer's protocol (5% non-fat dry milk in TBST or 5% BSA in TBST) for 1 h and incubated with a mouse p-P65 (Santa Cruz, 1:1,000), rabbit total P65 (Cell Signaling, 1:2,000), mouse FtH (Santa Cruz, 1:1,000), mouse FtL (Santa Cruz, 1:1,000), or goat FtL (Thermofisher or Santa Cruz, 1:1,000), followed by a peroxidase-conjugated goat anti-mouse or goat anti-rabbit or donkey anti-goat IgG antibody (Jackson ImmunoResearch Laboratories, 1:10,000). Horseradish peroxidase activity was detected using chemiluminescence (GE Healthcare) or KwikQuant detection system or LI-COR infrared Odyssey Imaging System. Membranes were stripped and reprobed with anti-GAPDH (Sigma-Aldrich, 1:10,000) or anti-β-actin (Sigma-Aldrich, 1:10,000) as a loading control. Densitometry analysis was performed using ImageStudio Lite and results were normalized to GAPDH or total P65 expression. Data were represented as fold change over controls.

### Blood Collection, Serum Preparation, and Analysis

Mice were anesthetized with 1.5–2% isoflurane and blood was collected by intra-cardiac puncture. Serum was isolated from blood after 30-min room temperature incubation and centrifugation. Aspartate transaminase activity (BioAssay Systems), HMGB1 (LSBio), iron (Abcam), hepcidin (eLabScience), NGAL (ENZO Life Sciences), hemopexin (Alpha Diagnostic), haptoglobin (Alpha Diagnostic), and ferritin (Kamiya Biomed) were measured in the serum according to manufacturers' protocols.

## Histology

Organs were collected and fixed in 10% neutral buffered formalin for 16 h prior to paraffin embedding. Paraffin sections were cut in to 4 μm sections, deparaffinized, and rehydrated using CitriSolv and isopropanol. Tissues were stained with hematoxylin and eosin stain using standard protocol. All images were acquired using a Leica DMI 6000B microscope (Leica Microsystems) and Leica Application Suite V4.2 software.

### RNA Sequencing Data Processing and Analysis

Raw sequencing (fastq) files were subjected to quality control analysis using FastQC (v0.11.5) [http://www.bioinformatics.babraham.ac.uk/projects/fastqc/] and using Trim Galore (v0.4.1) [http://www.bioinformatics.babraham.ac.uk/projects/trim_galore/], adapter sequences and poor quality reads were trimmed. Trimmed and cleaned sequence reads were aligned to mouse genome (GRCm38) using TopHat v2.1.0 (30) or STAR v2.5.3.a (31). The accepted BAM files from TopHat were sorted using samtools (Version: 1.3.1) (32) and reads aligning each annotated mouse gene were enumerated using HTSeq-count (33).

Differential expression analysis was performed using DESeq2 (34), following standard protocol [https://bioconductor.org/packages/release/bioc/vignettes/DESeq2/inst/doc/DESeq2.html]. Genes altered by absolute fold change of two or more with adjusted *p*-value < 0.01 were considered to be significantly differentially expressed [DEG]. DAVID (Database for Annotation, Visualization and Integrated Discovery) version 6.8 (35) was then used to conduct Gene Ontology (GO) and KEGG pathway enrichment analysis on DEG. Heatmap figures were generated in R 3.2.2 (https://cran.r-project.org/) using heatmap.2 function of gplots package [https://cran.r-project.org/web/packages/gplots/index.html].

## Quantification and Statistical Analysis

Data are represented as mean ± SEM. Unpaired 2-tailed *t*-test was used for comparisons between two groups. ANOVA and Tukey's multiple comparisons tests were used for comparisons between more than two groups. Survival significance was determined by Kaplan–Meier curve and log rank test. *P* < 0.05 were considered significant. All analysis was performed using GraphPad Prism 7.

## Data Availability

The accession numbers for the RNA sequencing data reported in this paper are Gene Expression Omnibus (GEO): GSE114078.

## Contact for Reagent and Resource Sharing

Further information and requests for resources and reagents should be directed to and will be fulfilled by the corresponding author, Subhashini Bolisetty (sbolisetty@uabmc.edu).

## Results

### Myeloid FtH Deficiency Prevents Multi-organ Failure and Mortality in Experimental Sepsis

**Figure 1:**
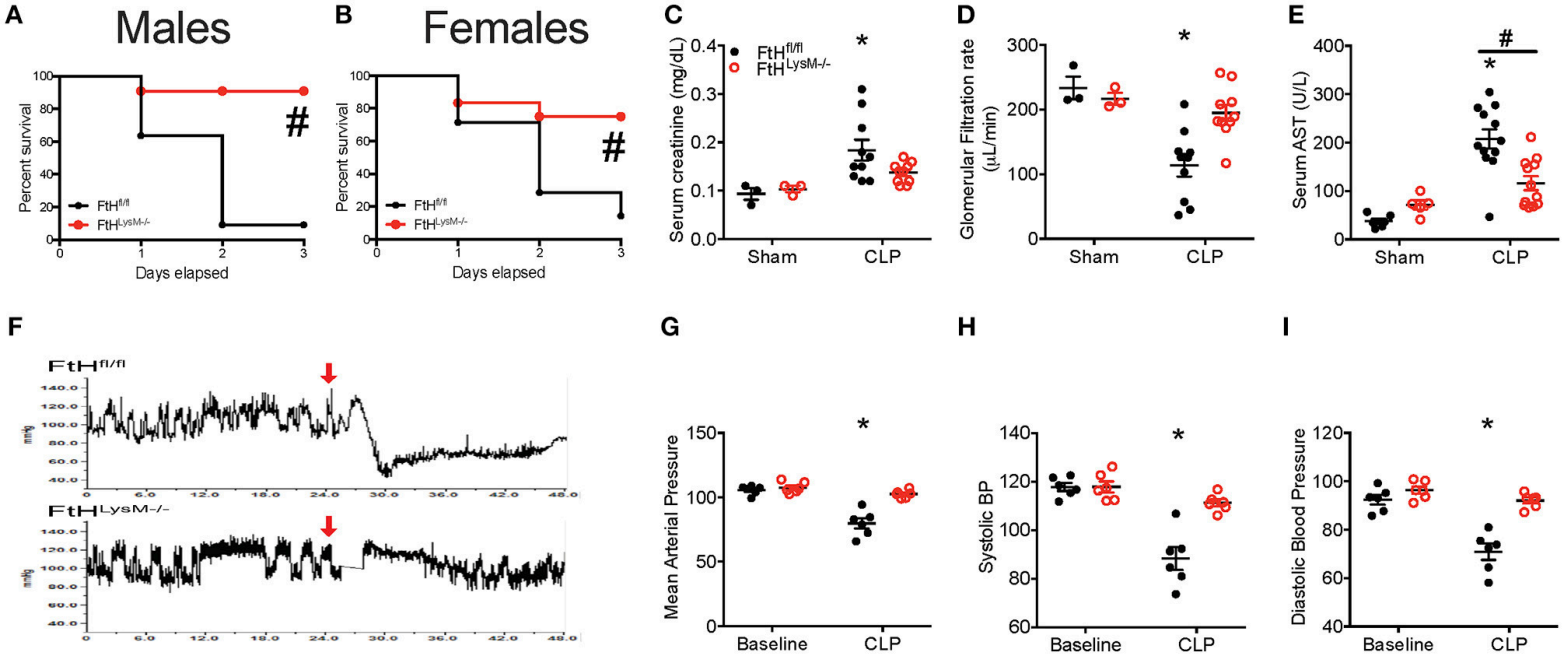
Myeloid FtH deficiency prevents multi-organ failure and mortality in experimental sepsis. **(A,B)** Percent survival of mice deficient in myeloid-specific FtH (FtH^LysM−/−^) and their floxed controls (FtH^fl/fl^) after cecal ligation puncture (CLP) surgery. **(A)** Males, *n* = 11 per group. **(B)** Females, *n* = 7–12 per group. Data are from three independent experiments; ^#^*p* < 0.05 vs. FtH^fl/fl^. **(C)** Serum creatinine levels were measured and expressed in milligrams per deciliter (mg/dL) as mean ± SEM 24 h after CLP or sham surgeries. Sham, *n* = 3 per group; CLP, *n* = 10 per group. *p < 0.05 vs. sham. **(D)** Transcutaneous glomerular filtration rate (GFR) was measured using FITC-sinistrin 24 h after CLP or sham surgery and expressed in microliters per minute (μL/min) as mean ± SEM. Sham, *n* = 3 per group; CLP, *n* = 10 per group. *p < 0.05 vs. sham. **(E)** Serum aspartate aminotransferase (AST) was measured 24 h after CLP surgery and expressed in units per liter (U/L) as mean ± SEM. Sham, *n* = 5–6 per group; CLP, *n* = 12 per group. *p < 0.05 vs. sham **(F–I)**. **(F)** Blood pressure was measured using radiotelemetry. Baseline measurements were recorded 24 h prior to CLP. Mice were monitored for 48 h. **(G)** Mean arterial pressure, **(H)** systolic, and **(I)** diastolic blood pressure were recorded 24 h following CLP in FtH^LysM−/−^ and FtH^fl/fl^ controls and expressed as mean ± SEM. *n* = 6 per group; *p < 0.05 vs. sham.

Following CLP, wild-type mice (FtH^fl/fl^) succumbed to significant mortality within 72 h following surgery whereas mice deficient in myeloid FtH (FtH^LysM−/−^) displayed significantly improved survival that was independent of gender (Figures 1A,B). Two important life-threatening attributes of sepsis are related to organ dysfunction and septic shock. Accordingly, we assessed the extent of organ injury following CLP and determined that myeloid FtH deficiency was associated with significantly improved preservation of renal function as evidenced by serum creatinine (Figure 1C) and transcutaneous glomerular filtration rate measurement (Figure 1D), lesser hepatic injury (AST, Figure 1E), and preserved circulatory status when compared to FtH^fl/fl^ mice (mean arterial pressure, systolic and diastolic blood pressure, Figures 1F–I). Additionally, structural damage to the lung and liver was less prominent, as confirmed by histological analyses (Figure S1). These findings suggest that FtH deficiency in the myeloid cells is associated with defense against sepsis induced organ dysfunction.

### Myeloid FtH Deficiency Dampens the Inflammatory Response in Sepsis

**Figure 2:**
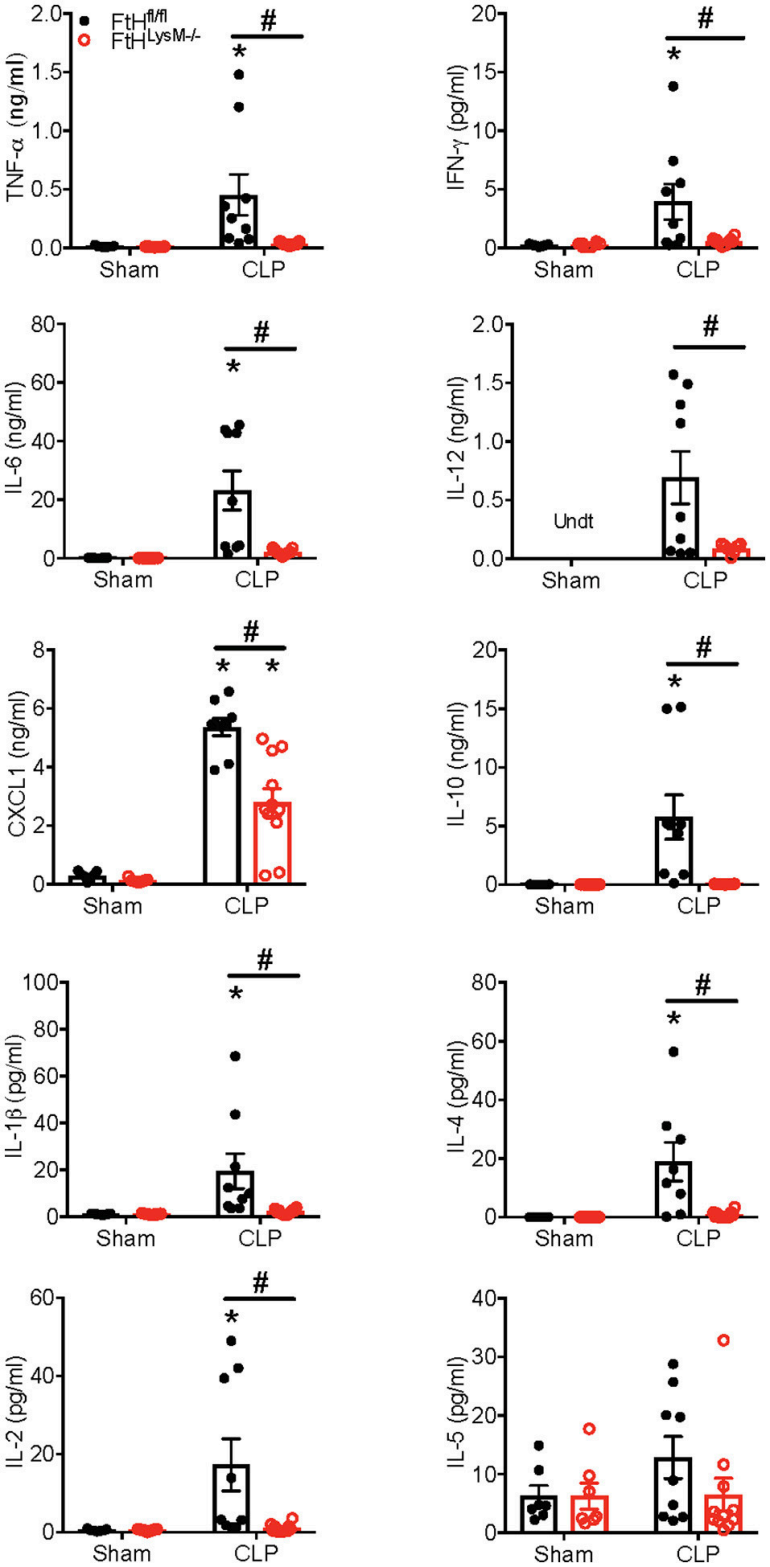
Myeloid FtH deficiency dampens the inflammatory response during sepsis Serum levels of tumor necrosis factor-α (TNF-α), interferon-γ (IFN-γ), interleukin-6 (IL-6), IL-12, chemokine ligand 1 (CXCL1), IL-10, IL-1β, IL-4, IL-2, and IL-5 were measured 24 h following sham or CLP surgery. Data are expressed in nanograms or picograms per milliliter (ng/mL or pg/mL) as mean ± SEM. Sham, *n* = 7 per group; CLP, *n* = 9–11 per group. Data are from two independent experiments. *p < 0.05 vs. sham, ^#^*p* < 0.05 vs. FtH^fl/fl^.

A dysregulated hyperinflammatory immune response is a hallmark of sepsis. In this context, we next assessed the levels of cytokines in the sera of mice that underwent sham or CLP surgery. In accordance with organ dysfunction in the FtH^fl/fl^ mice, 24 h after CLP surgery, there was a significant increase in the levels of cytokines that have been implicated in the pathogenesis of sepsis, including the pro-inflammatory cytokines, TNF-α, IFN-γ, IL-6, IL-12, CXCL1, IL-1β, and IL-2 (Figure 2). In contrast, FtH^LysM−/−^ mice displayed a non-significant increase in the levels of these cytokines when compared to sham controls. This response was also associated with a lack of significant induction in the anti-inflammatory cytokines such as IL-4 and IL-10 and had no apparent difference in the expression of IL-5, a cytokine that is predominantly produced by non-myeloid cells such as mast cells and T cells.

To assess the cross-talk between parenchymal and immune cells, we next analyzed several well-established markers of inflammation. Figure S2 depicts the expression profile of pro-inflammatory and anti-inflammatory cytokines and chemokines in the organs of myeloid FtH-deficient mice compared to their floxed wild-type controls that underwent sham or sepsis. Whether such regulation is contributed by the parenchymal cells or the resident myeloid cells within these organs is currently unknown. As expected, we found that splenic FtH induction following CLP was blunted in the FtH^LysM−/−^ mice (Figure S2). However, we did not find any association of FtH expression and cytokine levels in the organs following CLP. Expression of Heme oxygenase-1 (HO-1), an anti-oxidant enzyme with potent anti-inflammatory properties, was not significantly different following CLP in the organs of FtH^fl/fl^ compared to FtH^LysM−/−^ mice, suggesting that the protective effects of myeloid FtH deletion were independent of HO-1 (Figure S2).

**Figure 3:**
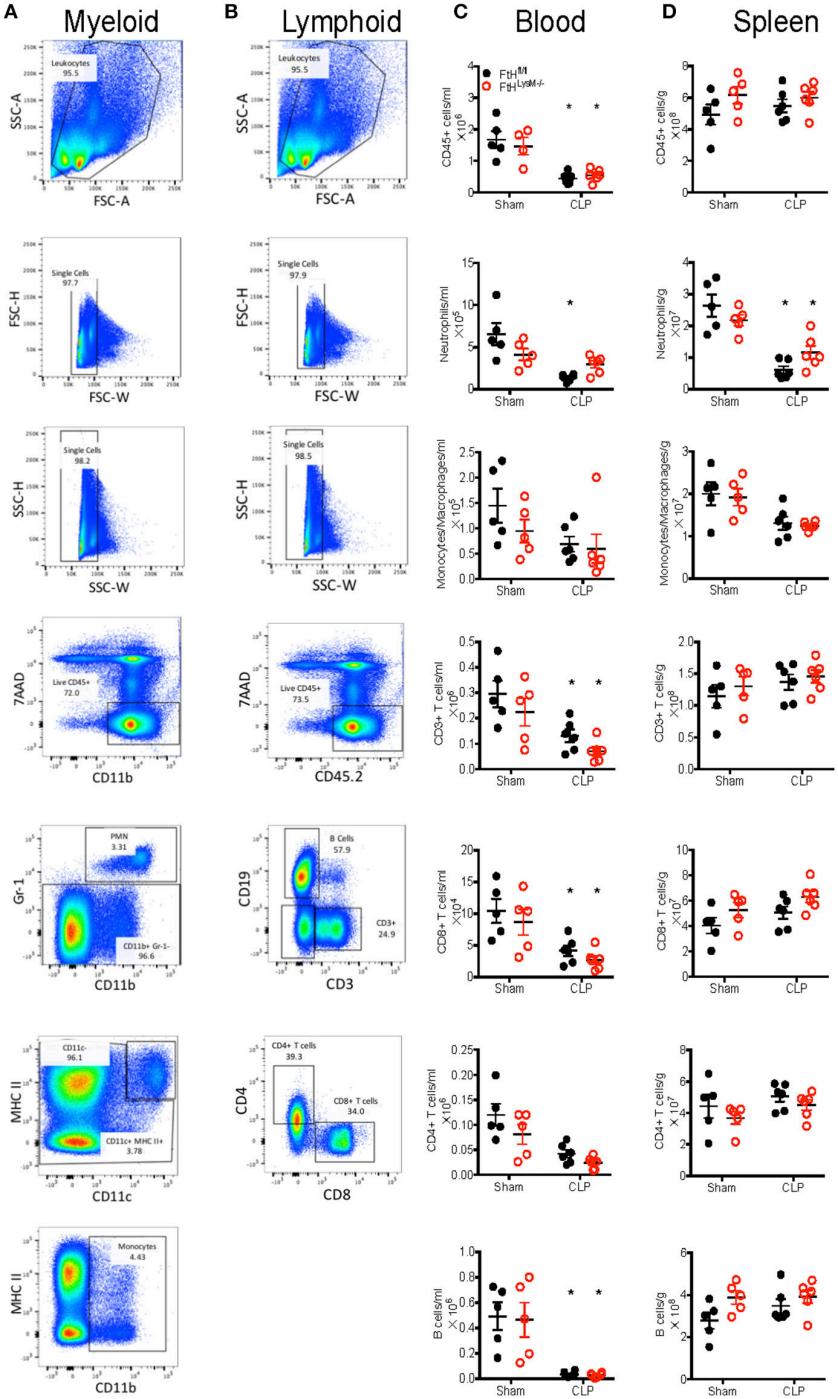
Myeloid FtH deficiency dampens the inflammatory response without altering the proportions of immune cell populations. **(A,B)** Representative flow cytometry histograms of **(A)** myeloid and **(B)** lymphoid panels from FtH^fl/fl^ sham control spleen demonstrating gating scheme and proportions of immune cell populations. **(C,D)** Quantification of number of cells in the **(C)** blood and **(D)** spleen 24 h after CLP. Data are from three independent experiments and are represented as number of cells per gram spleen (cells/g) or number of cells per ml blood as mean ± SEM. *n* = 4–5 per group. *p < 0.05 vs. sham.

We next characterized the profile of immune cell populations in the blood, spleen and lung to determine whether the protective effect of myeloid FtH deficiency is secondary to its role in regulating inflammation (Figures 3A,B and Figure S3A). Corroborating previous reports, we demonstrate that sepsis results in marked leukopenia in mice, leading to reduced CD45^+^ hematopoietic cells (Figure 3C) (36). More specifically, there was a significant reduction in the number of neutrophils in the blood and spleen following CLP in both FtH^fl/fl^ and FtH^LysM−/−^ mice. Interestingly, there was no difference in monocytes/macrophages (MM) (Figure 3C) or inflammatory MM (as defined by expression of Ly6C^hi^ and CX3CR1^+^CCR2^+^ macrophages) (Figure S3B). In support of previous reports, there is a significant reduction in peripheral as well as splenic T cells (CD3^+^), and specifically a decrease in cytotoxic CD8^+^ T cells following CLP in both the myeloid-ferritin knockout mice and controls (Figure 3C) (37–39). We also found a similar trend in the reduction of B cells following CLP (Figure 3C). In the spleen, we demonstrate a decrease in neutrophils and inflammatory macrophages (Ly6C^hi^ and CX3CR1^+^CCR2^+^), but no difference in the total macrophage, dendritic cell or lymphoid cell populations (Figure 3D and Figure S3C). We further demonstrate that while there was a significant increase in the proportion of macrophages, there was no significant difference in the neutrophil or dendritic cell populations in lungs of both the transgenic mice following CLP compared to sham (Figure S3D). Taken together, there was no significant difference in the lymphoid and myeloid populations following sepsis in the FtH^fl/fl^ vs. FtH^LysM−/−^ mice.

### FtH Expression Does Not Influence Phagocytosis or Bacterial Clearance

We next investigated whether the dampened cytokine response in the mice incompetent of FtH expression in the myeloid compartment was due to a difference in bacterial load or an inability of myeloid cells to detect, phagocytose, and clear bacteria. As evident from Figure S4A, there was no difference in the bacterial load in the peritoneal lavage of FtH^LysM−/−^ vs. FtH^fl/fl^ mice at 24 h following CLP. We also assessed the effect of myeloid FtH deficiency on bacterial clearance *in vivo*. Following intraperitoneal administration of chemiluminescent *E. coli* strain *Xen 14*, real-time IVIS imaging revealed no difference in bacterial clearance in FtH^LysM−/−^ vs. FtH^fl/fl^ mice (Figure S4B). These findings were further corroborated *in vitro* with infection of BMDMs with *Xen 14* (Figure S4C). Additionally, there was no significant difference in the phagocytic ability of myeloid FtH deficient BMDMs compared to wild-type BMDMs (Figure S4D). These results effectively rule out any attributable function of myeloid FtH in the context of bacterial recognition or phagocytosis by macrophages.

### Myeloid FtH Deletion Diminishes Response to Lipopolysacharide

**Figure 4:**
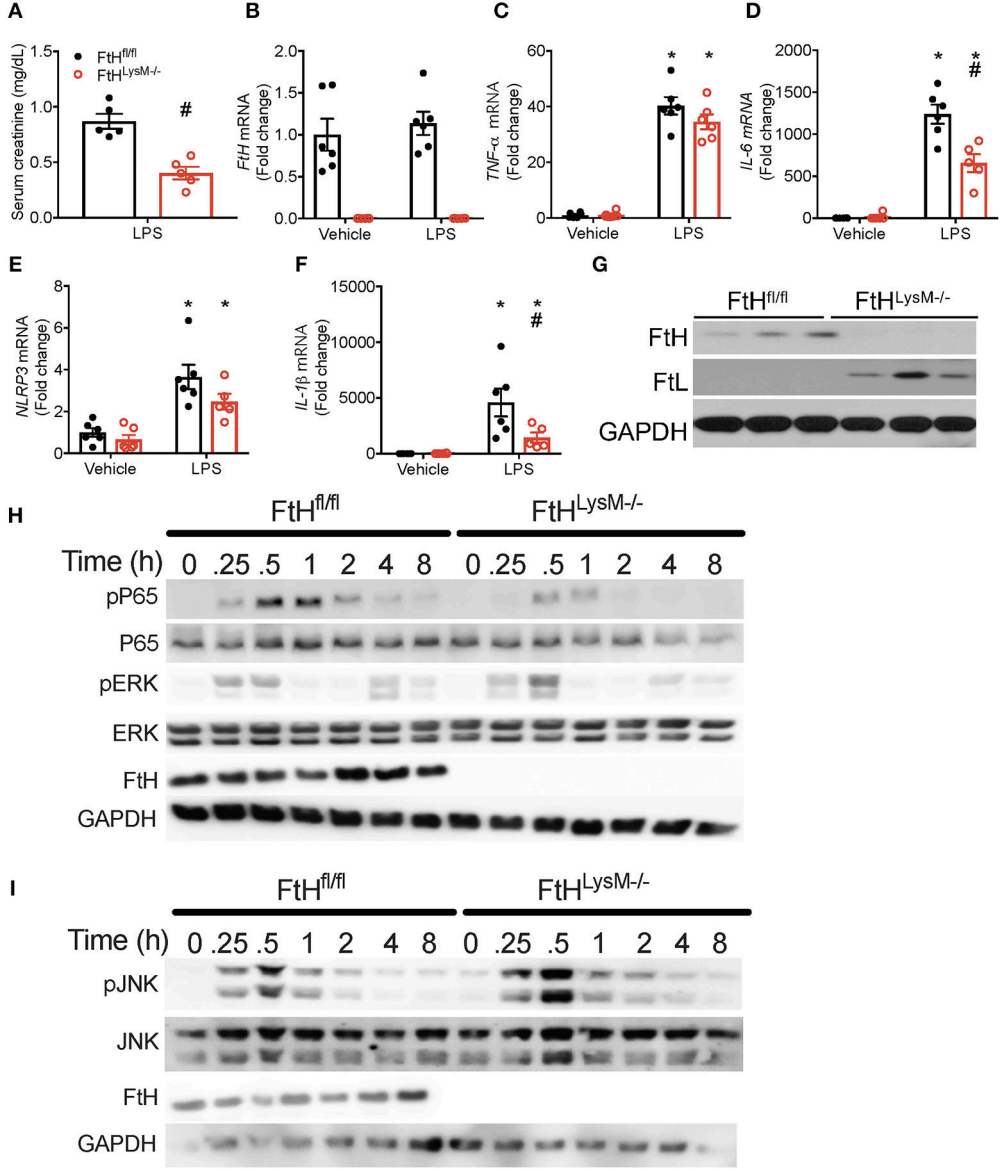
Myeloid FtH deletion diminishes response to lipopolysaccharide (LPS). **(A)** Serum creatinine levels were measured and expressed in mg/dL as mean ± SEM 24 h after LPS (8 mg/kg) or vehicle (normal saline). *n* = 5 per group; ^#^*p* < 0.05 vs. FtH^fl/fl^. **(B–G)** Real-time polymerase chain reaction (RT-PCR) analysis of mRNA expression of **(B)** *FtH*, **(C)** *TNF-*α, **(D)** *IL-6*, **(E)** *NACHT, LRR*, and *PYD domains-containing protein (NLRP3)*, and **(F)** *IL-1*β in bone marrow-derived macrophages (BMDMs) of FtH^fl/fl^ and FtH^LysM−/−^ mice that were treated with vehicle or LPS for 8 h. Data are analyzed and represented relative to GAPDH as mean ± SEM. *n* = 5–6 per group; *p < 0.05 vs. vehicle, ^#^*p* < 0.05 vs. FtH^fl/fl^. **(G)** Untreated BMDMs from FtH^fl/fl^ and FtH^LysM−/−^ mice were analyzed for expression of FtH and FtL. Membranes were stripped and reprobed for GAPDH expression to demonstrate equal loading. **(H,I)** BMDMs from FtH^fl/fl^ and FtH^LysM−/−^ mice were collected at 0, 0.25, 0.5, 1, 2, 4, and 8 h after LPS treatment and analyzed for expression of **(H)** phosphorylated p65 (pP65), total P65, phosphorylated extracellular signal-related kinase (pERK), FtH, and **(I)** phosphorylated c-Jun N-terminal kinase (pJNK), total JNK, and FtH. GAPDH was used as a loading control. Total ERK levels were examined in these lysates on a different membrane. Representative western blots from one out of three mice per genotype.

To further determine whether FtH expression regulates the response to lipopolysaccharide (LPS), a potent endotoxin that is implicated in the pathogenesis of gram-negative bacterial induced sepsis, we administered LPS to wild-type and myeloid FtH deficient mice. While FtH^fl/fl^ mice exhibited a significant loss of kidney function, FtH^LysM−/−^ mice were resistant to LPS-induced kidney injury (Figure 4A). Upon further investigation, we demonstrate that while LPS-induced IL-6 and IL-1β expression were significantly lower in FtH^LysM−/−^ macrophages compared to FtH^fl/fl^ macrophages, a non-significant trend was observed for TNF*-*α and NLRP3 mRNA expression (Figures 4B–F).

### Absence of FtH Led to Blunted Activation of NF-κB Following LPS

We next addressed whether the protective effects of FtH deletion are related to the regulation of nuclear factor kappa-light-chain enhancer of activated B cells (NF-κB; p65), a principal determinant of sepsis mediated injury (40). We demonstrate that LPS induced a time-dependent increase in the phosphorylation of p65 in FtH^fl/fl^ macrophages, an effect that was remarkably blunted in the FtH^LysM−/−^ cells (Figure 4H). We further evaluated macrophages for the expression of phosphorylated JNK and ERK, two key MAPK pathways that are activated following LPS stimulation. In corroboration with previous studies, absence of FtH led to an increase in LPS-induced phosphorylation of JNK (41) and ERK kinase (Figures 4H,I). Interestingly, we observed a bi-phasic activation of ERK following LPS stimulation, the latter activation is possibly driven by cytokines. As previously reported, lack of FtH in the macrophages from FtH^LysM−/−^ mice was associated with a compensatory increase in the levels of FtL (Figure 4G) (20).

### Serum Ferritin Is Significantly Higher in the Myeloid FtH Deficient Mice

**Figure 5:**
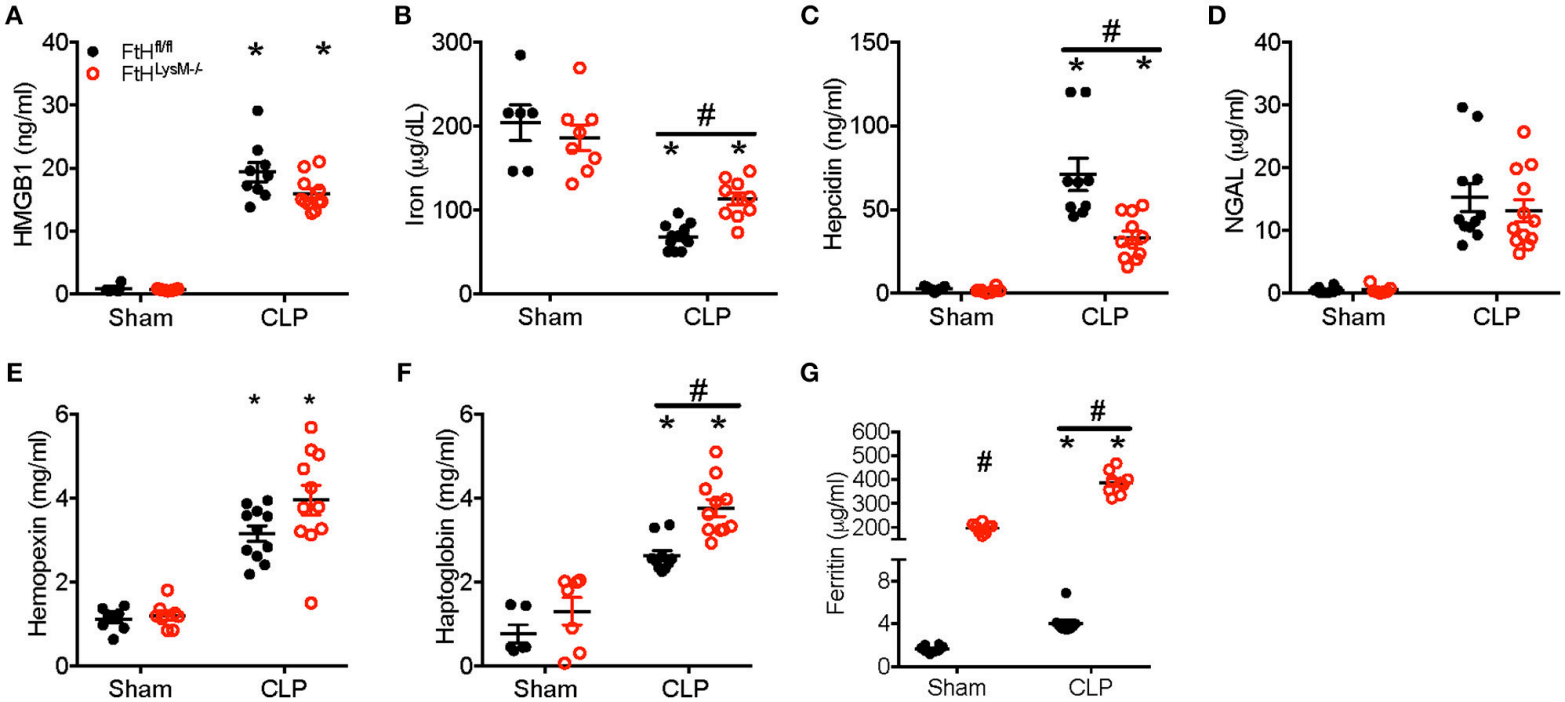
Serum ferritin is significantly higher in myeloid FtH deficient mice. Serum from FtH^fl/fl^ and FtH^LysM−/−^ mice 24 h after CLP and sham surgeries was collected and analyzed for **(A)** high mobility group box-1 (HMGB-1; ng/ml), **(B)** iron (μg/dL), **(C)** hepcidin (ng/ml), **(D)** neutrophil gelatinase associated lipocalin (NGAL; μg/ml), **(E)** hemopexin (mg/ml), **(F)** haptoglobin (mg/ml), and **(G)** ferritin (μg/ml). Data are expressed as mean ± SEM. *n* = 8–10 per group. *p < 0.05 vs. sham, ^#^*p* < 0.05 vs. FtH^fl/fl^.

At 24 h following CLP, there was no significant difference in the levels of high mobility group box 1 (HMGB1), a potential mediator of pathogenic events in sepsis (Figure 5A). HMGB1 is also implicated in leukocyte apoptosis and therefore may contribute to the significant decrease in leukocyte populations following CLP in both transgenic mice (Figure 3C). While serum iron levels were not different between sham-operated animals, CLP led to a significant reduction in serum iron levels in both the transgenic groups (Figure 5B). Additionally, serum iron levels were significantly higher in the FtH^LysM−/−^ mice that underwent CLP compared to FtH^fl/fl^ mice. As expected, hepcidin, an anti-microbial peptide, and a master regulator of iron homeostasis, was significantly higher in both the FtH^LysM−/−^ and FtH^fl/fl^ mice after CLP when compared to their respective sham controls. However, hepcidin levels were significantly lower in FtH^LysM−/−^ mice compared to FtH^fl/fl^ mice that underwent CLP (Figure 5C), which may explain the higher serum iron levels in FtH^LysM−/−^ mice following CLP. Furthermore, while neutrophil gelatinase-associated lipocalin (NGAL), another iron chelating protein with anti-bacterial properties, was significantly increased after CLP surgery, there was no significant difference attributed to myeloid FtH expression (Figure 5D). These results suggest that iron regulatory proteins, hepcidin and NGAL, do not account for the observed protective response in myeloid FtH deficient mice.

Hemolysis and resultant heme burden are implicated in the pathogenesis of sepsis (42). In fact, administration of hemopexin to quench free heme prevents sepsis injury in rodent models (42, 43). While we observed that hemopexin was significantly higher in mice that underwent CLP surgery, there was no influence of myeloid FtH on circulating hemopexin levels (Figure 5E). Haptoglobin, a protein that binds free hemoglobin is protective against sepsis pathogenesis and has been shown to decrease in the acute phase after sepsis (44, 45). We observed a significant reduction in serum haptogloboin levels 8 h after CLP, which was not different between FtH^fl/^ and FtH^LysM−/−^ mice (data not shown). However, we found significantly higher levels of haptoglobin at 24 h following CLP in the serum of FtH^LysM−/−^ when compared to FtH^fl/fl^ mice (Figure 5F).

We next investigated whether levels of serum ferritin, mainly comprised of ferritin light chain (FtL), were differentially regulated in the absence of myeloid FtH expression. Interestingly, we found that serum ferritin levels were significantly higher in the FtH^LysM−/−^ mice compared to FtH^fl/fl^ mice. Furthermore, we observed a significant increase in ferritin levels in mice that underwent CLP (irrespective of their myeloid FtH status) when compared to sham surgery (Figure 5G).

### FtL Confers Protection Against Sepsis-Induced Hyperinflammation and Organ Dysfunction

To investigate whether increased serum ferritin levels offered resistance to sepsis, we administered recombinant FtL to FtH^fl/fl^ mice, performed CLP surgery and measured the levels of inflammatory cytokines in the serum after 24 h (Table S1). We demonstrate a significant inhibitory effect on serum cytokine production in the presence of FtL following CLP surgery. Additionally, we also found that FtL administration led to significantly lesser hepatic injury as evidenced by AST (FtL CLP: 144 ± 9.4 vs. Vehicle CLP: 219 ± 16.8 U/L; *p* < 0.001). These findings suggest that FtL is associated with defense against sepsis-induced hyperinflammation and organ dysfunction.

### FtL Mitigates LPS-Induced MAPK and NF-κB Activation in Macrophages

**Figure 6:**
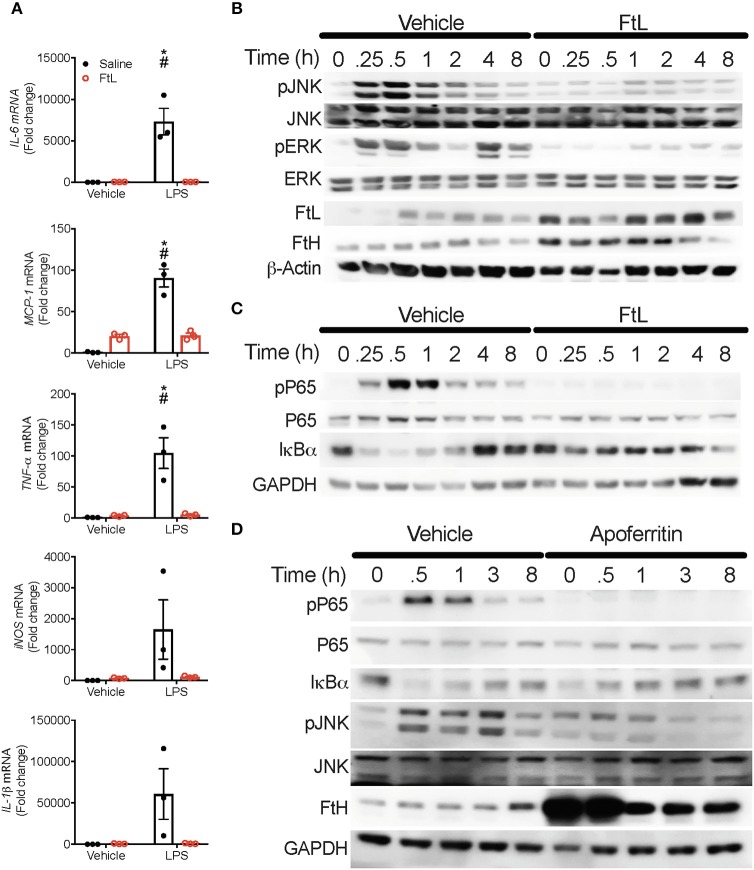
Ferritin light chain (FtL) mitigates LPS-induced MAPK and NF-κB activation. **(A)** BMDMs from FtH^fl/fl^ mice were pre-treated with FtL for 16 h followed by treatment with LPS or saline control. Cell lysates were collected 8 h later and analyzed for mRNA expression levels of *IL-6, monocyte chemoattractant protein-1 (MCP-1), TNF-*α*, inducible nitric oxide synthase (iNOS)*, and *IL-1*β, Data are normalized to GAPDH and fold change relative to saline vehicle are expressed as mean ± SEM. *n* = 3 from three independent experiments. *p < 0.05 vs. vehicle, ^#^*p* < 0.05 vs. LPS+FtL. **(B,C)** FtH^fl/fl^ BMDMs were pre-treated with FtL for 16 h, then washed and treated with LPS and collected at 0, 0.25, 0.5, 1, 2, 4, and 8 h. Cell lysates were analyzed for expression of **(B)** pJNK, total JNK, pERK, FtL, and FtH. β-actin was used as a loading control. Total ERK levels were examined in these lysates on a different membrane. **(C)** pP65, P65, and IκBα. GAPDH was used as a loading control. **(D)** FtH^fl/fl^ BMDMs were pre-treated with Apoferritin (ApoF) for 16 h, then washed and treated with LPS and collected at 0, 0.5, 1, 3, and 8 h. Cell lysates were analyzed for expression of pP65, P65, IκBα, pJNK, total JNK, and FtH. GAPDH was used as a loading control. Representative western blot from one out of three mice per treatment group.

Pre-treatment of BMDMs with FtL led to significantly lower induction of expression of inflammatory markers (*IL-6, IL-1*β*, TNF-*α*, MCP-1*) and *inducible nitric oxide synthase* (*iNOS*; Figure 6A). Pre-treatment with apoferritin, a holosphere that contains equal proportions of FtH and FtL, yielded similar results (Figure S5A). Pre-treatment with FtL led to reduced activation of LPS-mediated phosphorylation of JNK and ERK (Figure 6B). Additionally, pre-treatment with apoferritin also led to reduced phosphorylation of JNK when compared to vehicle treated cells (Figure 6D). These JNK activation data contrast our findings in LPS treated FtH^LysM−/−^ macrophages (Figure 4I), suggesting a cumulative effect of FtH and FtL expression on mitigating JNK activation. LPS treatment activates the NF-κB pathway (Figure 4), therefore, we determined whether FtL regulates NF-κB activation. Interestingly, we demonstrate that pre-treatment with FtL or apoferritin mitigates LPS-induced phosphorylation of p65 (Figures 6C,D). In addition, we found that LPS-mediated degradation of IκBα was markedly less pronounced in macrophages that were pre-treated with FtL or apoferritin (Figures 6C,D). Of note, we found that treatment with FtL led to an increase in expression of FtH expression. Also, phosphorylated p65 levels were significantly lower in the spleens of FtH^LysM−/−^ mice compared to FtH^fl/fl^ mice following CLP (Figures S5C–E). Taken together these data suggest that FtL diminishes NF-κB activation and downstream inflammatory response and confers resistance to sepsis.

To mimic the polymicrobial flora of our *in vivo* model, we prepared cecal slurry from FtH^fl/fl^ mice as previously described (27). Our data demonstrated that pre-treatment with apoferritin led to an abated immune response in the presence of cecal slurry (Figure S5B). Specifically, induction of *IL-6, MCP-1, TNF-*α*, and IL-1*β was markedly lower in the presence of apoferritin. Corroborating our previous findings, we demonstrate that apoferritin treatment induced expression of *iNOS* (Figure S5B) (20).

### Alteration of Gene Expression Profile of Blood Leukocytes Following Sepsis

**Figure 7:**
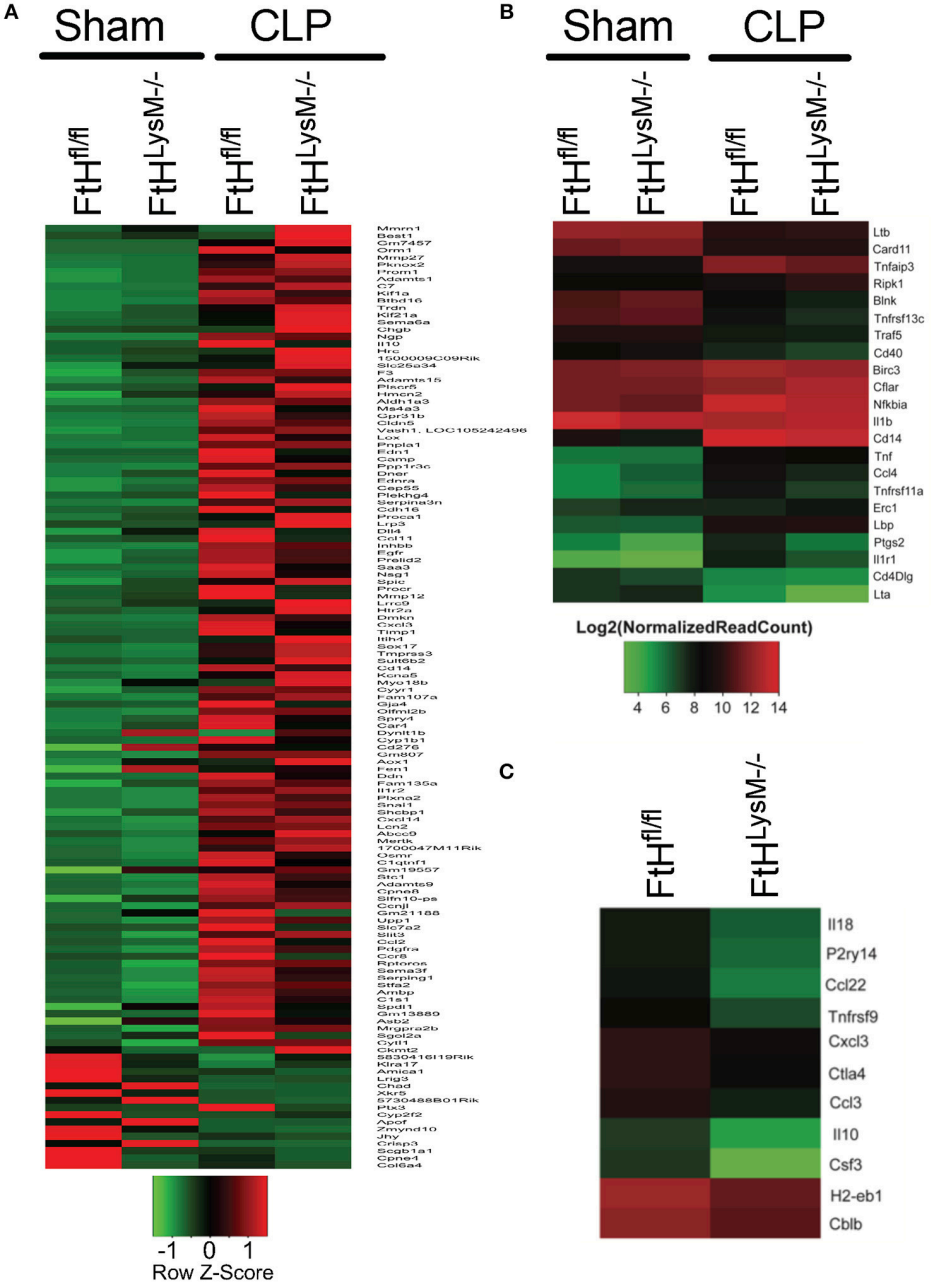
Alteration of gene expression profile of blood leukocytes following sepsis. RNA sequencing was performed on blood leukocytes isolated from FtH^fl/fl^ and FtH^LysM−/−^ mice following CLP or sham surgery. **(A)** Hierarchical clustering of genes that are significantly altered (*p* < 0.01) between leukocytes from the transgenic mice at 8 h following surgery. Heatmap displays z-transformed expression values. *n* = 3 per group. **(B)** NF-κB signaling pathway genes differentially expressed in FtH^fl/fl^ and FtH^LysM−/−^ CLP compared to FtH^fl/fl^ sham. The differentially expressed genes were identified based on absolute fold change of two or more and adjusted *p* value < 0.01. *n* = 3 per group. **(C)** Genes associated with “immune response” biological process [GO: 0006955] down-regulated or differentially expressed in FtH^LysM−/−^ CLP compared to FtH^fl/fl^ CLP. The differentially expressed genes were identified based on absolute fold change of two or more and adjusted *p* < 0.01. *n* = 3 per group.

We next performed transcriptomic analyses of blood leukocytes from FtH^LysM−/−^ and FtH^fl/fl^ mice following sham or CLP surgery. The heatmap shown in Figure 7A highlights the significant (*p* < 0.01) differential expression of transcripts following CLP in both the transgenic mice compared to sham controls. Corroborating our cytokine data, we found that CLP led to enrichment of biological processes that are associated with an immune response (Tables S2, S3). Interestingly, in comparison with FtH^fl/fl^, CLP induced transcriptomic changes in the FtH^LysM−/−^ mice demonstrated a clear pattern of dampened immune response. These include downregulation of genes associated with processes such as the inflammatory response, the response to LPS and cell proliferation (Table S2). Additionally, genes associated with processes such as oxidant detoxification and regulation of NF-κB signaling were upregulated in the myeloid FtH deficient mice (Table S3). Hierarchical clustering of the NF-κB-dependent gene pathway identified several genes that were differentially regulated in the FtH^LysM−/−^ mice (Figure 7B). Additionally, there was a clear difference in the expression levels of genes associated with the immune response between the FtH^LysM−/−^ and FtH^fl/fl^ mice that underwent CLP (Figure 7C).

## Discussion

In this study we investigated whether deletion of FtH in the myeloid cells impacts CLP-induced inflammation and organ injury. Our findings revealed that deletion of FtH was associated with significant disease protection against sepsis. This was evident by lower mortality, better preserved kidney function and blood pressure, lesser hepatic injury and serum cytokine levels, and decreased organ inflammation in myeloid FtH deficient mice. We found similar bacterial killing capabilities ruling out these processes as underlying mechanisms for the observed differences. Importantly, our results showed a significantly higher level of serum ferritin in both sham and CLP groups of FtH^LysM−/−^ mice compared to their floxed counterparts. Since the macrophages are the main source of circulating ferritin (16), the higher serum ferritin was likely due to consequent compensatory overexpression of FtL in myeloid cells lacking FtH. Our *in vitro* and *in vivo* FtL administration studies indicate that prior exposure to FtL provides significant resistance to the septic process by mitigating overproduction of cytokines involved in the pathogenesis of sepsis. Our results indicate a major inhibitory action by ferritin on NF-κB activation and its downstream effects. RNA-Seq analyses of circulating leukocytes validate the paramount importance of serum ferritin in inhibition of the NF-κB pathway identifying novel functional properties of this protein.

The exact role of iron in the pathogenesis of sepsis and how iron regulatory proteins are involved in this process remains under investigation (46–49). In this context, little is known about the role of serum ferritin and its association with sepsis, particularly its pathogenesis and outcomes. Immediately following infection, a cascade of events shapes an environment known as state of hypoferremia that is intended to limit availability of iron to the infectious microbes (50, 51). This process is primarily driven by synthesis of hepcidin which in turn leads to endocytosis and degradation of ferroportin (52). Other pathways that are modulated by robust elevation of cytokines further solidify this strategy (53). The overall effect is decreased iron absorption and increased iron retention within reticuloendothelial cells. Serum ferritin (mainly FtL) is used to measure body iron stores in healthy individuals but its increased level in response to infection and inflammation limits its utility (17, 54). The perplexing evolutionary purpose of this increase in circulating iron poor ferritin during inflammatory processes is yet to be fully divulged.

To understand the adaptation to sepsis induced inflammation, we examined iron regulatory proteins that are beneficial during sepsis. Our findings do not support such protection to be mediated by hepcidin or NGAL, as neither of these proteins were significantly elevated in FtH^LysM−/−^ mice compared to floxed controls. However, we found that serum ferritin was strikingly high in both groups of FtH^LysM−/−^ mice undergoing sham or CLP surgeries. Further analysis revealed high levels of FtL expression in macrophages of FtH^LysM−/−^ mice that underscores a compensatory mechanism to FtH gene deletion. Since the major source of circulating ferritin is FtL (16), we postulated that this elevated serum ferritin may play a role in inflammation during sepsis. In support of this premise, we found a mitigated inflammatory response to LPS in macrophages with overexpression of FtL to compensate for FtH deletion. Moreover, when wild-type macrophages were treated with apoferritin or recombinant FtL (all devoid of iron) we found a similar pattern of resistance toward LPS or bacterial exposure that was evident by lesser induction of inflammatory markers, suggesting a less profound, and restrained response to infectious stimuli. As expected, we found a significant increase in intracellular FtL following administration of apoferritin and FtL. The cardinal pathway that mediates the response of inflammatory cells to infectious or injurious stimuli is the NF-κB pathway (40, 55). Our *in vitro* findings disclose a striking blockade of this pathway with preconditioning cells to various forms of ferritin. Also, a recent study demonstrated that transfection of macrophages with FtL led to reduced nuclear accumulation of p65 subunit after LPS treatment (56). Given the importance of the spleen as a secondary lymphoid organ and its role in orchestrating adaptive immune responses, we validated the abrogation of the NF-κB pathway as a central regulator of the phenotypes observed in FtH^LysM−/−^ mice. Moreover, taking advantage of the unbiased analysis of the transcriptome of circulating leukocytes by RNA-Seq, we further corroborate the unique signature of downregulation of the NF-κB pathway and genes associated with “immune response” biological process in FtH^LysM−/−^ mice following CLP induced sepsis.

Our understanding of iron metabolism and role of ferritin in various disease models is evolving. In a study published by Lipinski et al. in 1991, it was reported that tissue ferritin administration protects against *E. coli*-induced sepsis in mice (57). Furthermore, Weis et al. recently described how FtH mediated metabolic adaptation markedly improves disease tolerance to sepsis (58). It is important to note that these studies demonstrate how FtH induction plays a protective role during sepsis by inhibiting the iron-mediated oxidative inhibition of liver glucose-6-phosphatase and consequently sustain adequate gluconeogenesis. In contrast, our findings predominantly emphasize the significance of circulating FtL in modulating inflammatory cells toward a restrained response to infection.

It is recognized that a well-established anti-inflammatory cytokine, IL-10, is also robustly induced during inflammation to provide a counterbalance to an otherwise unchecked deleterious immune response. Similar pathways have been well-described in other physiological contexts such as the rapid induction of the anti-coagulation pathway during activation of the coagulation cascade. For the first time, our findings provide insight into the elusive nature and function of circulating ferritin. These findings suggest an important role for serum ferritin in regulating a controlled and measured response to infection in order to minimize a dysregulated and heightened injurious inflammatory process that is central to the detrimental outcomes of sepsis. These findings introduce a novel function for circulating FtL that establishes its role as an immunomodulatory cytokine. It must be noted that these results neither preclude utility of serum FtL as a marker of iron stores under physiological circumstances, nor do they rule out the association that has been described between high levels of circulating ferritin and worse outcomes in various inflammatory diseases (14). In contrast, our results suggest that similar to IL-10, the increment in the level of circulating ferritin should be regarded as an ongoing struggle to limit the body's inflammatory reaction toward injurious stimuli. Notably, elevated serum ferritin is a common finding in patients with end stage renal disease who require renal replacement therapy. Such elevated serum ferritin levels do not always mirror the iron status of these patients (17). Furthermore, a significant number of these patients dialyze via a permcath that distinctively predisposes them to bacteremia (59–61). Hence, future studies will need to investigate the rate of bacteremia/sepsis in these patients and their outcomes compared to patients with normal ferritin levels.

In summary, we provide novel findings that identify the NF-κB pathway as a main target of circulating ferritin to establish a restrained response to infection. These results will provide a novel platform for future studies to better understand the pathogenesis of sepsis and novel targets for potentially new strategies to challenge the significant burden of sepsis induced morbidity, mortality and substantial health care expenditure.

## Author Contributions

AZ and SB formulated the hypothesis, designed the study, performed most of the experiments, and wrote the manuscript. TH and RB performed and analyzed data from the flow cytometry experiments. SV and DC analyzed the RNA sequencing data and performed pathway analysis. WF performed radiotelemetry studies. LB performed the glomerular filtration measurements. LB, SB, and AZ performed bacterial clearance and phagocytosis experiments, all the remaining experiments were performed by AZ, KM, SE, and SB. PA and MP generated recombinant ferritin light chain protein. PA and JB provided scientific input. All authors read and approved the manuscript.

### Conflict of Interest Statement

The authors declare that the research was conducted in the absence of any commercial or financial relationships that could be construed as a potential conflict of interest.

## Supplementary Material

Figure S1 Myeloid FtH deficiency prevents multi-organ failure and mortality in experimental sepsis. Hematoxylin and eosin (HandE) staining of liver and lung tissue from FtH^fl/fl^ and FtH^LysM−/−^ mice 24 h following CLP. Structural damage to the liver is evidenced by steatosis, necrosis, and ballooning degeneration of hepatocytes. Histologic examination of the lung demonstrates interstitial edema and infiltration of immune cells. Scale bar = 100 μm.

Figure S2 Myeloid FtH deficiency dampens the inflammatory response following sepsis. Spleen, liver, lung, and kidney tissues were analyzed for gene expression levels of *IL-6, IL-10, MCP-1, TNF-*α*, iNOS, HO-1*, and *FtH* in FtH^fl/fl^ and FtH^LysM−/−^ mice 24 h after CLP. Data are expressed as fold change relative to GAPDH as mean ± SEM. Sham, *n* = 4 per group; CLP, *n* = 8 per group. ^*^*p* < 0.05 vs. sham, ^#^*p* < 0.05 vs. FtH^fl/fl^.

Figure S3 Myeloid FtH deficiency dampens the inflammatory response without altering the proportions of immune cell populations. **(A)** Representative flow cytometry histograms of monocyte/macrophage populations from FtH^fl/fl^ sham control demonstrating gating scheme and proportions of immune cell populations. **(B–D)** Quantification of number of cells in the **(B)** blood, **(C)** spleen, and **(D)** lung 24 h after CLP. Data are represented as number of cells per gram kidney (cells/g) as mean ± SEM. *n* = 4–5 per group. ^*^*p* < 0.05 vs. sham.

Figure S4 FtH expression does not influence phagocytosis or bacterial clearance. **(A)** Colony forming units (CFU) in FtH^fl/fl^ and FtH^LysM−/−^ mice peritoneal fluid 24 h after CLP. Data are expressed in CFU/mL as mean ± SEM. *n* = 9–12 per group. **(B)** *In vivo* bacterial clearance was assessed by IVIS bioluminescence. Mice were infected with *E. coli* Xen14 (10^6^) intraperitoneally and imaged. Data are expressed in photons per second (photons/sec) as mean ± SEM. *n* = 7–8 per group; ^*^*p* < 0.05 vs. 0H. **(C)** *In vitro* bacterial killing was measured in mouse bone marrow-derived macrophages infected with *E. coli* Xen14 (MOI 10 and 100). Data are expressed as mean ± SEM in A.U. *n* = 6 per group. **(D)** *In vitro* phagocytic activity was assessed using pHrodo Red *E. coli* BioParticles using flow cytometry. Median fluorescence intensity is expressed as arbitrary units (A.U.) ± SEM. *n* = 3 per group.

Figure S5 FtL confers protection against sepsis by mitigating NF-κB activation. **(A)** FtH^fl/fl^ BMDMs were treated with LPS with or without pre-treatment with apoferritin or vehicle control for 16 h. Cells were collected after 8 h and analyzed for expression of *FtH, IL-6, TNF-*α*, NLRP3*, and *IL-1*β *mRNA*. Data are normalized to GAPDH and fold change relative to controls are expressed as mean ± SEM. *n* = 6 per group. ^*^*p* < 0.05 vs. vehicle, ^#^*p* < 0.05 vs. LPS+Apoferritin. **(B)** FtH^fl/fl^ BMDMs were treated with cecal slurry (C.S.) after pretreatment with apoferritin or saline control. Cells were collected after 8 h and analyzed for expression of *MCP-1, IL-6, TNF-*α*, IL-1*β, and *iNOS*. Data are normalized to GAPDH and fold change relative to controls are expressed as mean ± SEM. *n* = 5 per group. **(C–E)** Spleens from FtH^fl/fl^ and FtH^LysM−/−^ mice 24 h after CLP were analyzed for expression of pP65, P65, and FtH. GAPDH was used as a loading control. **(D)** pP65 was normalized to P65 and **(E)** FtH was normalized to GAPDH and densitometric values were expressed as A.U. *n* = 7 per group. ^#^*p* < 0.05 vs. FtH^fl/fl^.

Table S1 Serum cytokine profile of FtH^fl/fl^ + FtL compared to FtH^fl/fl^.

Table S2 Downregulated genes in FtH^LysM−/−^ compared to FtH^fl/fl^ during sepsis.

Table S3 Upregulated genes in FtH^LysM−/−^ compared to FtH^fl/fl^ during sepsis.

Table S4 Primers for Real-time PCR analysis.
