# Supplementary figures and images for: Ferritin Light Chain Confers Protection Against Sepsis-Induced Inflammation and Organ Injury
Source: Front Immunol. 2019 Feb 4;10:131. doi: 10.3389/fimmu.2019.00131 (PMC6371952; doi:10.3389/fimmu.2019.00131)

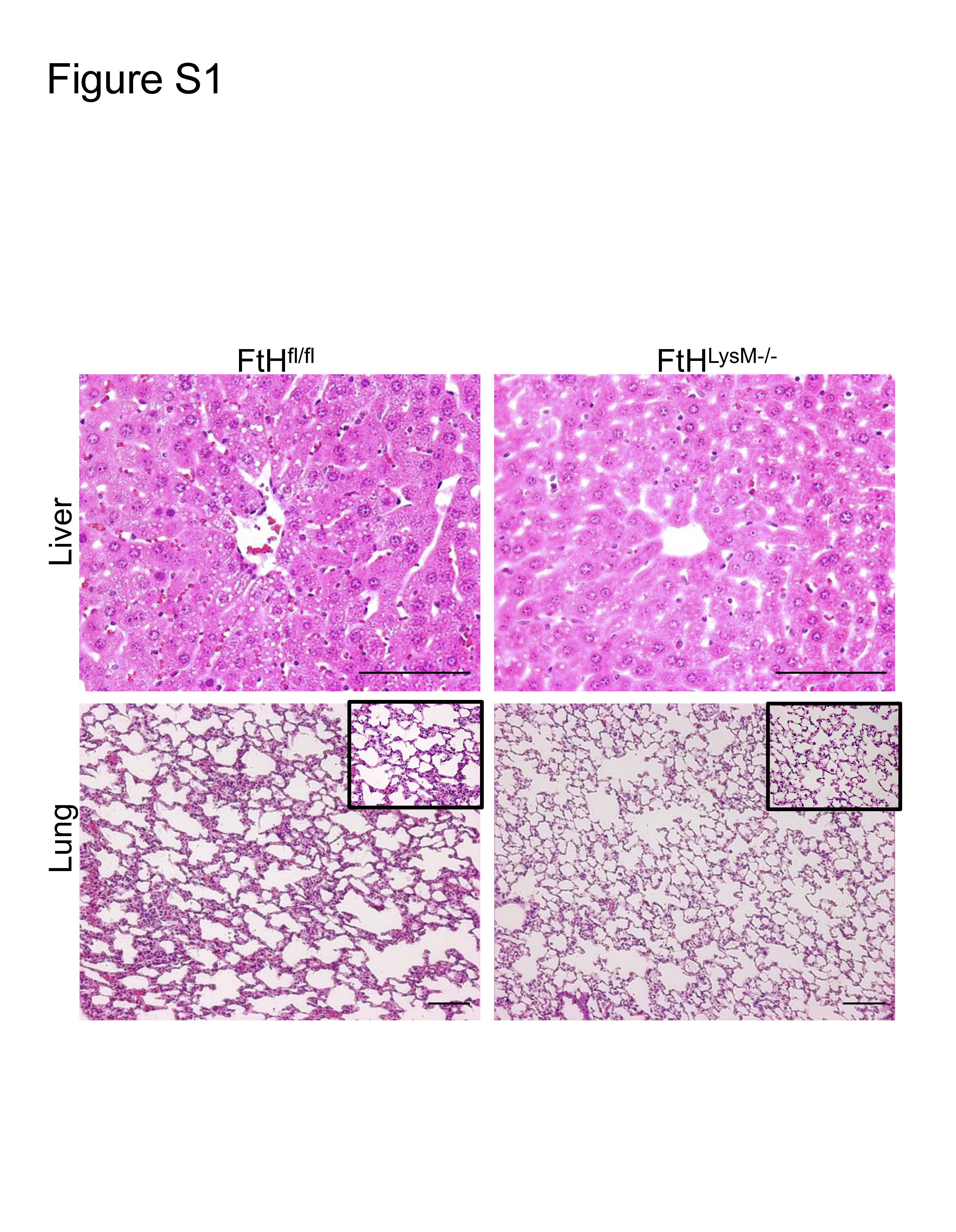

Supplement: Figure S1 — Myeloid FtH deficiency prevents multi-organ failure and mortality in experimental sepsis. Hematoxylin and eosin (HandE) staining of liver and lung tissue from FtHfl/fl and FtHLysM−/− mice 24 h following CLP. Structural damage to the liver is evidenced by steatosis, necrosis, and ballooning degeneration of hepatocytes. Histologic examination of the lung demonstrates interstitial edema and infiltration of immune cells. Scale bar = 100 μm. [file Image_1.TIFF]

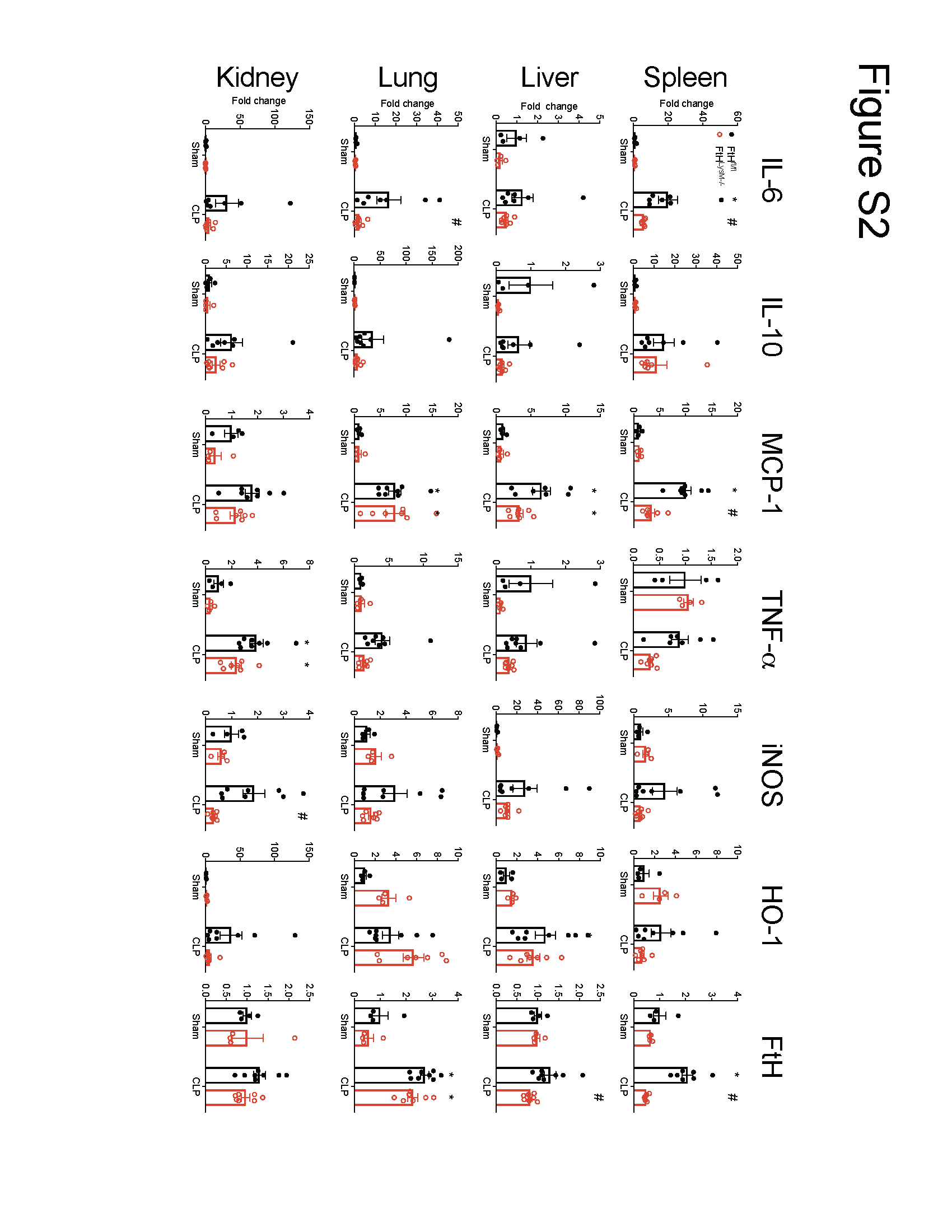

Supplement: Figure S2 — Myeloid FtH deficiency dampens the inflammatory response following sepsis. Spleen, liver, lung, and kidney tissues were analyzed for gene expression levels of IL-6, IL-10, MCP-1, TNF-α, iNOS, HO-1, and FtH in FtHfl/fl and FtHLysM−/− mice 24 h after CLP. Data are expressed as fold change relative to GAPDH as mean ± SEM. Sham, n = 4 per group; CLP, n = 8 per group. *p < 0.05 vs. sham, #p < 0.05 vs. FtHfl/fl. [file Image_2.TIFF]

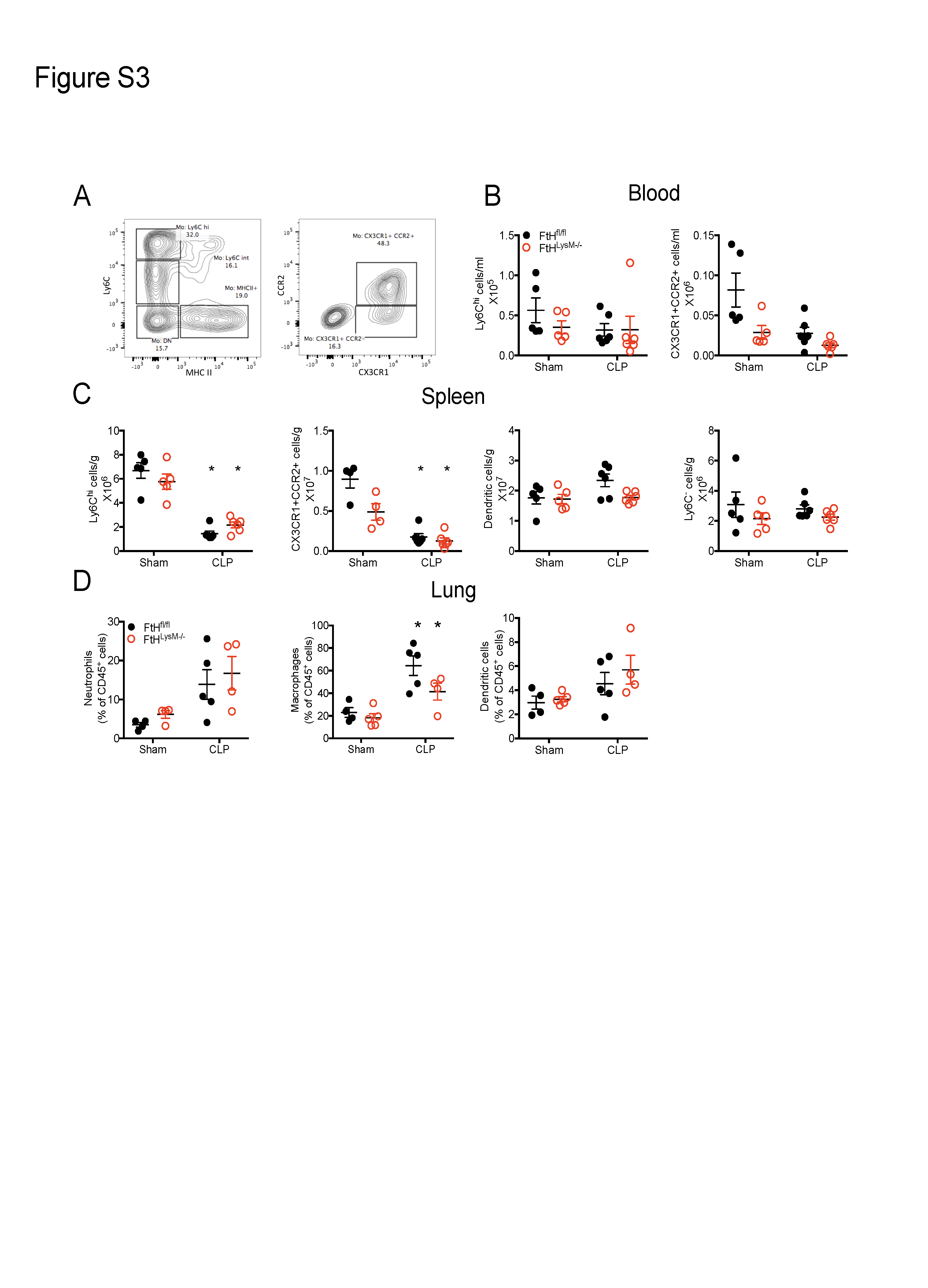

Supplement: Figure S3 — Myeloid FtH deficiency dampens the inflammatory response without altering the proportions of immune cell populations. (A) Representative flow cytometry histograms of monocyte/macrophage populations from FtHfl/fl sham control demonstrating gating scheme and proportions of immune cell populations. (B–D) Quantification of number of cells in the (B) blood, (C) spleen, and (D) lung 24 h after CLP. Data are represented as number of cells per gram kidney (cells/g) as mean ± SEM. n = 4–5 per group. *p < 0.05 vs. sham. [file Image_3.TIFF]

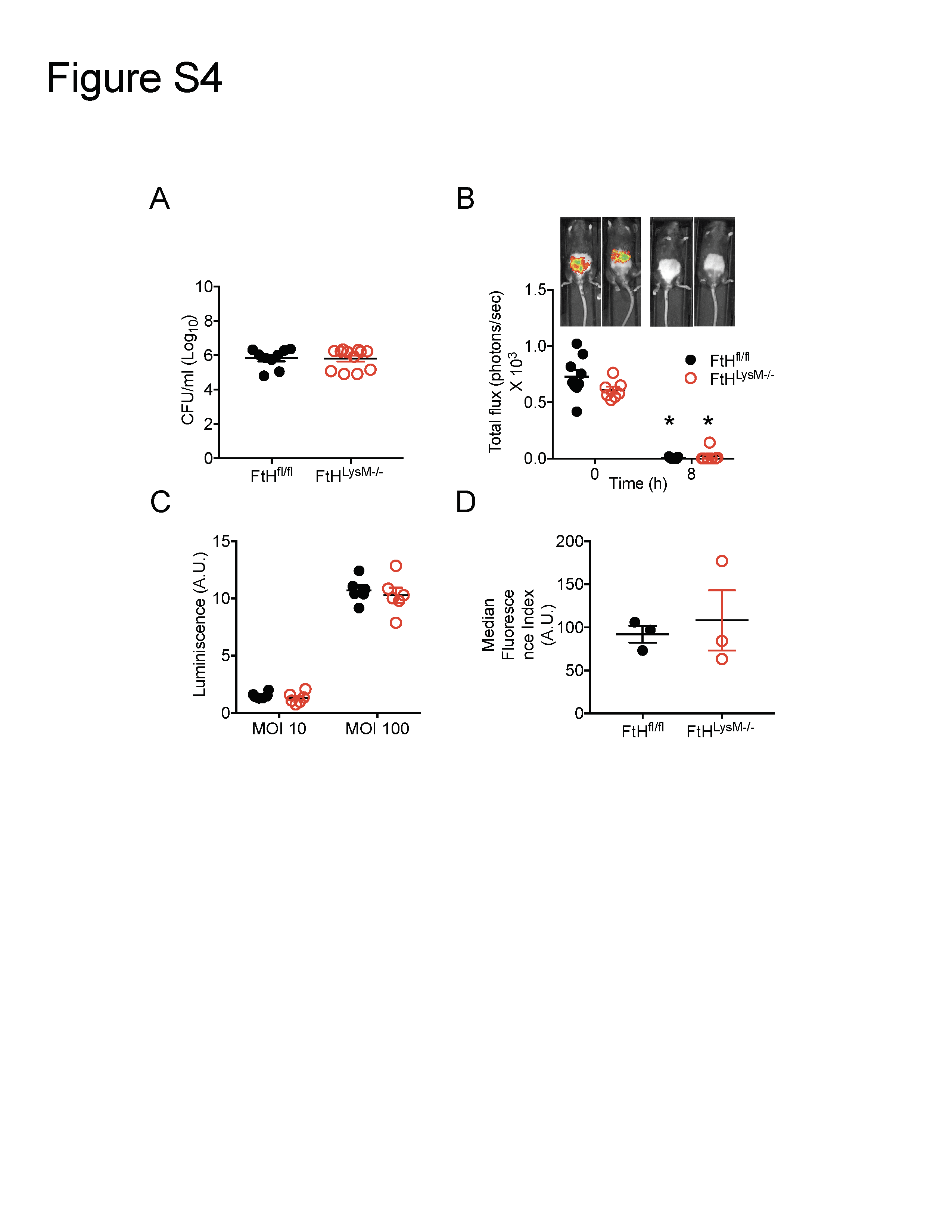

Supplement: Figure S4 — FtH expression does not influence phagocytosis or bacterial clearance. (A) Colony forming units (CFU) in FtHfl/fl and FtHLysM−/− mice peritoneal fluid 24 h after CLP. Data are expressed in CFU/mL as mean ± SEM. n = 9–12 per group. (B) In vivo bacterial clearance was assessed by IVIS bioluminescence. Mice were infected with E. coli Xen14 (106) intraperitoneally and imaged. Data are expressed in photons per second (photons/sec) as mean ± SEM. n = 7–8 per group; *p < 0.05 vs. 0H. (C) In vitro bacterial killing was measured in mouse bone marrow-derived macrophages infected with E. coli Xen14 (MOI 10 and 100). Data are expressed as mean ± SEM in A.U. n = 6 per group. (D) In vitro phagocytic activity was assessed using pHrodo Red E. coli BioParticles using flow cytometry. Median fluorescence intensity is expressed as arbitrary units (A.U.) ± SEM. n = 3 per group. [file Image_4.tiff]

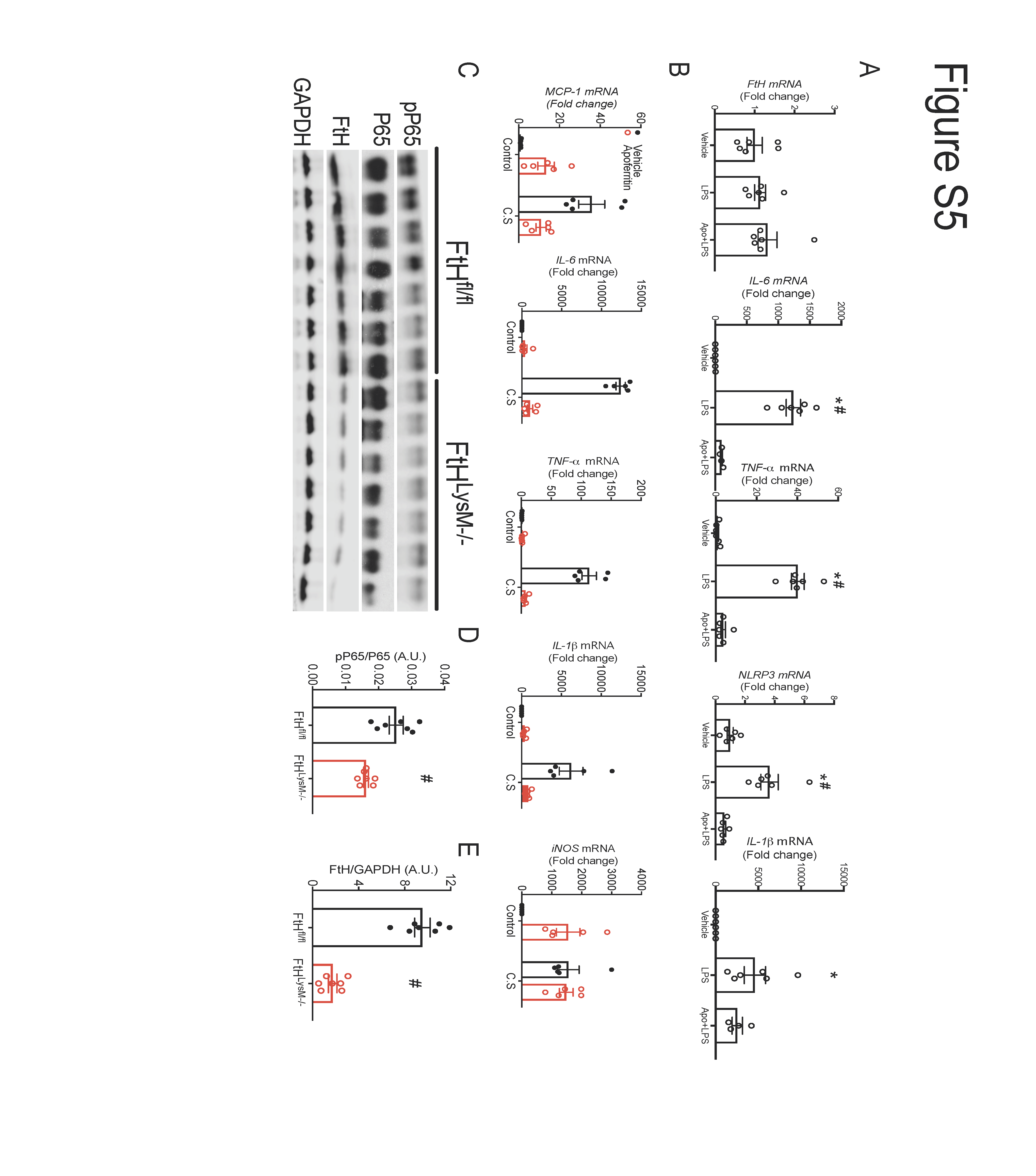

Supplement: Figure S5 — FtL confers protection against sepsis by mitigating NF-κB activation. (A) FtHfl/fl BMDMs were treated with LPS with or without pre-treatment with apoferritin or vehicle control for 16 h. Cells were collected after 8 h and analyzed for expression of FtH, IL-6, TNF-α, NLRP3, and IL-1β mRNA. Data are normalized to GAPDH and fold change relative to controls are expressed as mean ± SEM. n = 6 per group. *p < 0.05 vs. vehicle, #p < 0.05 vs. LPS+Apoferritin. (B) FtHfl/fl BMDMs were treated with cecal slurry (C.S.) after pretreatment with apoferritin or saline control. Cells were collected after 8 h and analyzed for expression of MCP-1, IL-6, TNF-α, IL-1β, and iNOS. Data are normalized to GAPDH and fold change relative to controls are expressed as mean ± SEM. n = 5 per group. (C–E) Spleens from FtHfl/fl and FtHLysM−/− mice 24 h after CLP were analyzed for expression of pP65, P65, and FtH. GAPDH was used as a loading control. (D) pP65 was normalized to P65 and (E) FtH was normalized to GAPDH and densitometric values were expressed as A.U. n = 7 per group. #p < 0.05 vs. FtHfl/fl. [file Image_5.TIFF]
