# Supplementary material for: Ferritin Light Chain Confers Protection Against Sepsis-Induced Inflammation and Organ Injury
Source: Front Immunol. 2019 Feb 4;10:131. doi: 10.3389/fimmu.2019.00131 (PMC6371952; doi:10.3389/fimmu.2019.00131)
Supplement: Table S1 — Serum cytokine profile of FtHfl/fl + FtL compared to FtHfl/fl. [file Table_1.pdf]

Table S1. Serum cytokine profile of $FtH^{fl/fl}$ + FtL compared to $FtH^{fl/fl}$

**Cytokine (pg/ml)**	**FtL** (n = 5)	**Vehicle CLP** (n = 11)	**FtL CLP** (n = 11)	**p value (FtL CLP vs. FtL)**	**p value (FtL CLP vs. Vehicle CLP)**
IFNγ	1.06±0.14	4.58±0.84	0.56±0.16	ns	<0.0001
IL-1β	2.2±0.2	34.35±7.37	3.098±1.1	ns	0.0249
IL-2	1.81±0.69	27.18±5.07	0.47±0.11	ns	0.0004
IL-4	1.18±0.47	13.16±4.09	0.44±0.17	ns	0.0031
IL-5	2.3±0.57	12.9±1.78	4.3±1.67	ns	0.0306
IL-6	76.3±2.7	28905±3191	1701±514	ns	0.0009
IL-10	102±9.2	3075±711	292±125	ns	0.023
IL-12	159±27	1229±330	49.4±11.4	ns	0.0007
CXCL1	143.97±23	5466±238	1306±376	ns	0.0014
TNFα	25.45±5.6	517±83	60.87±24	ns	0.0076
