# Supplementary material for: Ferritin Light Chain Confers Protection Against Sepsis-Induced Inflammation and Organ Injury
Source: Front Immunol. 2019 Feb 4;10:131. doi: 10.3389/fimmu.2019.00131 (PMC6371952; doi:10.3389/fimmu.2019.00131)
Supplement: Table S2 — Downregulated genes in FtHLysM−/− compared to FtHfl/fl during sepsis. [file Table_2.pdf]

Table S2. Downregulated genes in $FtH^{LysM-/-}$ compared to $FtH^{fl/fl}$ during sepsis

GO ID	Biological processes	# Genes	P Value
44130	negative regulation of growth of symbiont in host	5	7.45E-06
6955	immune response	11	1.09E-05
7049	cell cycle	15	4.29E-05
6882	cellular zinc ion homeostasis	4	2.01E-04
42127	regulation of cell proliferation	8	6.90E-04
51301	cell division	10	7.30E-04
1878	response to yeast	3	0.001133
6260	DNA replication	6	0.001226
6954	inflammatory response	9	0.001788
7059	chromosome segregation	5	0.002663
9411	response to UV	4	0.00348
8285	negative regulation of cell proliferation	9	0.003516
50832	defense response to fungus	3	0.005298
60707	trophoblast giant cell differentiation	3	0.005935
30889	negative regulation of B cell proliferation	3	0.005935
42493	response to drug	8	0.006543
82	G1/S transition of mitotic cell cycle	4	0.007567
51412	response to corticosterone	3	0.008044
31668	cellular response to extracellular stimulus	3	0.008812
32496	response to lipopolysaccharide	6	0.009148
10243	response to organonitrogen compound	3	0.00961
30593	neutrophil chemotaxis	4	0.010147
6270	DNA replication initiation	3	0.01044
6829	zinc II ion transport	3	0.01219
1701	in utero embryonic development	7	0.013186
