# Supplementary material for: Ferritin Light Chain Confers Protection Against Sepsis-Induced Inflammation and Organ Injury
Source: Front Immunol. 2019 Feb 4;10:131. doi: 10.3389/fimmu.2019.00131 (PMC6371952; doi:10.3389/fimmu.2019.00131)
Supplement: Table S3 — Upregulated genes in FtHLysM−/− compared to FtHfl/fl during sepsis. [file Table_3.pdf]

Table S3. Upregulated genes in $FtH^{LysM-/-}$ compared to $FtH^{fl/fl}$ during sepsis

GO ID	Biological processes	# Genes	P Value
98869	cellular oxidant detoxification	4	4.79E-06
48821	erythrocyte development	5	5.71E-05
6936	muscle contraction	5	6.51E-04
8285	negative regulation of cell proliferation	11	9.71E-04
35556	intracellular signal transduction	11	0.001318
15671	oxygen transport	3	0.002681
30097	hemopoiesis	5	0.004824
1655	urogenital system development	3	0.006931
7420	brain development	7	0.007476
60216	definitive hemopoiesis	3	0.008746
45666	positive regulation of neuron differentiation	5	0.009064
7010	cytoskeleton organization	5	0.00937
46777	protein autophosphorylation	6	0.015222
35335	peptidyl-tyrosine dephosphorylation	3	0.015296
34395	regulation of transcription from RNA polymerase II promoter in response to iron	2	0.023539
8588	release of cytoplasmic sequestered NF-kappaB	2	0.023539
45214	sarcomere organization	3	0.024862
30036	actin cytoskeleton organization	5	0.026357
51209	release of sequestered calcium ion into cytosol	3	0.034533
48873	homeostasis of number of cells within a tissue	3	0.036266
43124	negative regulation of I-kappaB kinase/NF-kappaB signaling	3	0.038032
10999	regulation of eIF2 alpha phosphorylation by heme	2	0.038926
6468	protein phosphorylation	10	0.03956
38083	peptidyl-tyrosine autophosphorylation	3	0.041661
55072	iron ion homeostasis	3	0.043522
