# Supplementary material for: Ferritin Light Chain Confers Protection Against Sepsis-Induced Inflammation and Organ Injury
Source: Front Immunol. 2019 Feb 4;10:131. doi: 10.3389/fimmu.2019.00131 (PMC6371952; doi:10.3389/fimmu.2019.00131)
Supplement: Table S4 — Primers for Real-time PCR analysis. [file Table_4.pdf]

Table S4. Primers for Real-time PCR analysis

Gene	Forward primer	Reverse Primer
MCP-1	5’-ACTCACCTGCTGCTACTCAT-3’	5’-CTACAGCTTCTTTGGGACA-3’
IL-1β	5’-TGGGCCTCAAAGGAAAGA-3’	5’-GGTGCTGATGTACCAGTT-3’
IL-6	5’-CTGCAAGAGACTTCCATCCAG-3’	5’AGTGGTATAGACAGGTCTGTTGG-3
TNF-α	5’-ACGGCATGGATCTCAAAGAC-3’	5’-AGATAGCAAATCGGCTGACG-3’
iNOS	5’-CCAAGCCCTCACCTACTTCC-3’	5’-CTCTGAGGGCTGACACAAGG-3’
IL-10	5’-GCTCTTACTGACTGGCATGAG-3’	5’-CGCAGCTCTAGGAGCATGTG-3’
NLRP3	5’-CTGTGTGTGGGACTGAAGCAC-3’	5’-GCAGCCCTGCTGTTTCAGCAC-3’
FtH	5’-CCATCAACCGCCAGATCAAC-3’	5’-GCCACATCATCTCGGTCAAA-3’
HO-1	5’-GGTGATGGCTTCCTTGTACC-3’	5’-AGTGAGGCCCATACCAGAAG-3’
GAPDH	5’-ATCATCCCTGCATCCACT-3’	5’-ATCCACGACGGACACATT-3’
